# Body dysmorphia in a 23 year old patient with obsessive compulsive disorder: a case report

**DOI:** 10.1192/j.eurpsy.2024.1304

**Published:** 2024-08-27

**Authors:** P. Setién Preciados, E. Arroyo Sánchez, C. Díaz Mayoral

**Affiliations:** Servicio de Psiquiatría, Hospital Universitario Príncipe de Asturias, Alcalá de Henares, Spain

## Abstract

**Introduction:**

Body dysmorphic disorder (BDD) was considered an anxiety disorder in the DSM-IV, but in the DSM-V was added to the obsessive-compulsive and related disorders category. BDD is a psychiatric disorder characterised by an excessive, persistent, and distressful preoccupation with a perceived defect in appearance. These perceived defects are slight and are unnoticed by others. People with BDD usually have poor insight and are preoccupied with a perceived physical defect which causes them to check on it repeatedly. This leads to an impairment in psychosocial functioning, depression, and an increase in suicide risk.

**Objectives:**

Review how dismorphophobia (BDD) and obsessive compulsive syndrome intersect, the differences they present in symptomatology, prevalence and treatment.

**Methods:**

Presentation of a patient’s case and review of existing literature, in regards to body dysmorphic syndrome and its similarities and differences with respect to obsessive compulsive syndrome.

**Results:**

There are common features between both disorders, which are genetic overlap, physical past traumatic events, sex ratio, trait of perfectionism and body image disturbance.

Studies have found the prevalence of BDD in patients with OCD in a large patient sample was 8.7% to 15% compared to 3% in non-OCD.

The risk of comorbidity of OCD-BDD is three times higher in samples with a primary diagnosis of BDD compared to those with a primary diagnosis of OCD with 27.5% and 10.4%, respectively.

BDD as well as OCD must be managed with pharmacological and psychotherapy treatment. A selective serotonin reuptake inhibitor is the recommended first-line medication for BDD, even if appearance beliefs are delusional in nature.

Serotonin reuptake inhibitor (SRI) doses and trial durations are similar to those used for OCD; higher doses and a longer treatment trial are recommended than those typically used for depression and most other disorders. Cognitive-behavioral therapy that is specifically tailored to BDD is the psychosocial treatment of choice. Simply treating BDD as if it were OCD is not recommended.

**Conclusions:**

There are limitations included a restricted number of studies overall, an absence of studies comparing biological parameters, and the frequent inclusion of participants with comorbid body dysmorphic disorder and obsessive-compulsive disorder. The current nosological status of body dysmorphic disorder is somewhat tenuous and requires further investigation, with particular focus on dimensional, biological and aetiological elements.

**Disclosure of Interest:**

None Declared

